# Uterine Fibroids, Perceived Stress, and Menstrual Distress: a Key Role of Heavy Menstrual Bleeding

**DOI:** 10.1007/s43032-022-01126-3

**Published:** 2022-12-05

**Authors:** Silvia Vannuccini, Sara Clemenza, Emanuele Cassioli, Eleonora Rossi, Giovanni Castellini, Valdo Ricca, Felice Petraglia

**Affiliations:** 1 Department of Experimental, Clinical and Biomedical Sciences, University of Florence, Careggi University Hospital, Florence, Italy; 2 Psychiatry Unit, Department of Health Sciences, University of Florence, Florence, Italy

**Keywords:** Dysmenorrhea, Heavy menstrual bleeding, Menstruation, Menstrual distress, Stress, Uterine fibroids

## Abstract

Uterine fibroids (UFs) are the most common benign tumors in women of reproductive age, frequently associated with pain symptoms and heavy menstrual bleeding (HMB), leading to impaired quality of life. Thus, the aim of the study was to evaluate the global perception of stress and the menstrual distress in patients with UFs. A cross-sectional observational study was conducted on a group (*n* = 69) of fertile age women with UFs compared to age-matched controls, by administering two questionnaires: the perceived stress scale (PSS) and the Menstrual Distress Questionnaire (MEDI-Q). The PSS, MEDI-Q Total Score and 3 subscales—menstrual symptoms (MS), menstrual symptoms distress (MSD), and menstrual specificity index (MESI)—were evaluated. Patients with UFs showed higher PSS than controls (18.5 ± 5.0 vs. 13.8 ± 5.0, *p* < 0.001) and PSS values were very high in those with HMB, severe dysmenorrhea, and impaired social and working life. Patients with UFs also showed significantly higher score for MEDI-Q Total Score (16.51 ± 12.99 vs. 10.86 ± 12.36) (*p* < 0.01) as well as for the subscales MSD (2.54 ± 1.07 vs. 1.57 ± 0.98) (*p* < 0.001) and MESI (0.76 ± 0.30 vs 0.60 ± 0.39) (*p* < 0.05). The menstrual distress was associated to being uncomfortable about uterine bleeding; in fact, MEDI-Q Total Score was significantly higher in women with HMB compared to those with moderate/normal bleeding. UF characteristics (number, type, and size) did not correlate with perceived stress or menstrual distress. In conclusion, women with UFs have significantly higher levels of perceived stress and menstrual distress than controls and HMB plays a major role in determining such conditions.

## Introduction

Uterine fibroids (UFs) are the most common benign tumors in women of childbearing age and the leading cause of hysterectomy among premenopausal women [1–3]. Patients with UFs suffer a variety of different symptoms: abnormal uterine bleeding (heavy menstrual bleeding (HMB) and irregular bleeding), pain (dysmenorrhea and dyspareunia) symptoms associated with fibroid growth (pelvic pressure, increased urinary frequency, constipation, and abdominal bloating), and infertility [4, 5]. The presence of these symptoms is not constant in all patients and some cases may be asymptomatic; however, a number of studies has revealed that UFs have a relevant impact on women’s health and on quality of life (QoL) [6–9]. Indeed, patients with UFs may show an impairment of sexuality, self-image, social life, emotional and physical well-being, and work productivity [8]. Notably, UFs are associated with levels of disability similar to those of other chronic diseases: affected women show low scores in the domain of vitality and social functioning, highlighting the significant psychosocial burden of UFs [10]. Moreover, a significant improvement in QoL after both surgical (myomectomy, hysterectomy) [11–14], medical [15–19], or physical treatments [20, 21] is reported.

Chronic stress plays a significant role in determining a decrease of QoL because it causes psychological wear and tear to some extent [22], and distress defines a maladaptive response to a stressor, mirrored by an impairment of QoL [23]. Limited data are available on distress in patients with UFs [24], which may contribute to stress-related depression [25].

Thus, the present study is aimed at evaluating the global perception of stress and the menstrual distress in patients with UFs compared to a control group and their possible correlation with clinical symptoms and UF characteristics.

## Material and Methods

An observational cross-sectional study was conducted in a group of patients with UFs (*n* = 69, age range between 29 and 49 years) referred to the Obstetrics and Gynecology Division of the University of Florence (Careggi University Hospital) for a consultation for UFs between September 2020 and June 2021. The inclusion criteria for the study population were reproductive age, first diagnosis of UFs by ultrasound or magnetic resonance imaging (MRI), and no current medical treatment for UFs. An age-matched group of fertile age women (*n* = 69) without gynecological disorders referring to the hospital for routine gynecological checkup was used as control group. In patients with UFs and in controls data on menarche and characteristics of the menstrual cycle (time interval between menstruation and amount and duration of menstrual bleeding) were collected (Table 1). Furthermore, in patients with UFs information on location, number and size of UFs, pain symptoms, and bleeding pattern were reported (Table 2).

**Table 1:** Menstrual cycle characteristics of UFs and control groups

	UFs (*n* = 69)	Controls (*n* = 69)	*p*
Age of menarche (years)	12.5 ± 1.6	12.4 ± 2.0	0.785
Menstrual cycle length (days)	27.7 ± 7.5	28.8 ± 6.1	0.165
Regular menstrual cycle	46 (66.7%)	53 (76.8%)	0.256
Menstrual bleeding			
Light	6 (8.7%)	12 (17.3%)	0.001
Moderate	19 (27.5%)	42 (60.8%)	
Heavy	44 (63.8%)	15 (21.7%)	
Duration of menstrual bleeding	6.3 ± 4.5	5.1 ± 2.1	0.0001

**Table 2:** UF characteristics (*n* = 69)

Number of fibroids	
Single	26.6%
Multiple	73.4%
Type of fibroids	
Subserosal	67.4%
Intramural	79.3%
Submucosal	34.8%
Size	
< 3 cm	6.6%
4–6 cm	64.1%
> 7 cm	30.3%
Clinical presentation	
At least one symptom	57.9%
Pelvic pain	35%
Dysmenorrhea	22.5%
Dyspareunia	7.5%
Urinary disorders	10%
Pelvic pressure	15%
HMB	63.8%
Infertility	12.5%

Two different questionnaires, the Menstrual Distress Questionnaire (MEDI-Q) [26] and the Italian version of the perceived stress scale (PSS-10) [27, 28], were administered to both cases and control. The PSS-10 is a widely used psychological instrument for measuring the global perception of stress. It is a 10-item questionnaire that evaluates the degree to which an individual has perceived life as unpredictable, uncontrollable, and overloading over the previous month before the interview. The questions ask about feelings and thoughts during the last month. In each case, respondents are asked how often they felt a certain way on a five-point scale from “never” (= 0) to “very often” (= 4). The PSS score is then obtained by summing across all scale items. The score is divided into four categories (range, 0–40 points): 0–6, low level of stress; 7–19, medium level of stress; 20–25, high level of stress; and ≥ 26, very high level of stress.

The MEDI-Q aims to assess and score the distress related to menstruation across the last 12 months. The instrument investigates 25 items, covering the following areas: pain, discomfort, psychic/cognitive changes, gastrointestinal symptoms, and changes in physiological functions. The MEDI-Q Total Score evaluates the global menstruation-related distress, whereas 3 additional subscales investigate the number of menstrual symptoms (MS) that exacerbate during menstruation, the average distress related to menstrual symptoms (menstrual symptom distress, MSD), and the specificity of menstrual symptoms (menstrual specificity index, MESI) [26].

The study protocol was approved by the Ethics Committee of the Institution (n.14558_oss approved on 28 May 2019). A written informed consent was requested for participation to the study. Exclusion criteria were overt menopause, non-Italian native-speakers, illiteracy, or inability to provide informed consent.

### Statistical Analysis

The data extrapolated from the questionnaires and baseline characteristics of the cases and controls were entered in an electronic database and analyzed using the software SPSS (Statistical Package for Social Science) (IBM SPSS Statistics 23, IBM Corporation). A descriptive analysis was conducted with the evaluation of position measures (mean, median) and dispersion indices (standard deviation, range) for the quantitative variables. The binomial variables were described by calculating the absolute and percentage frequencies. Pearson’s chi-square test or Fisher’s exact test was used to compare the qualitative variables, depending on the size of the sample. For the quantitative variables, the t-test or the Mann–Whitney test was used, depending on whether or not the distribution of the data was normal. One-way ANOVA was also used to compare MEDI-Q Total Score, MS, MSD, and MESI according to menstrual bleeding (normal, moderate, and heavy). Regression analysis was used to test the association between MEDI-Q scores and UF characteristics and clinical presentation. Statistical significance was considered in case of *p* < 0.05.

## Results

Regarding the characteristics of menstrual cycle, patients with UFs reported more frequently HMB (63.8% vs. 21.7%, *p* = 0.001) and prolonged menstruation than controls (6.3 ± 4.5 vs. 5.1 ± 2.1, *p* = 0.0001) (Table 1).

**Fig. 1:**
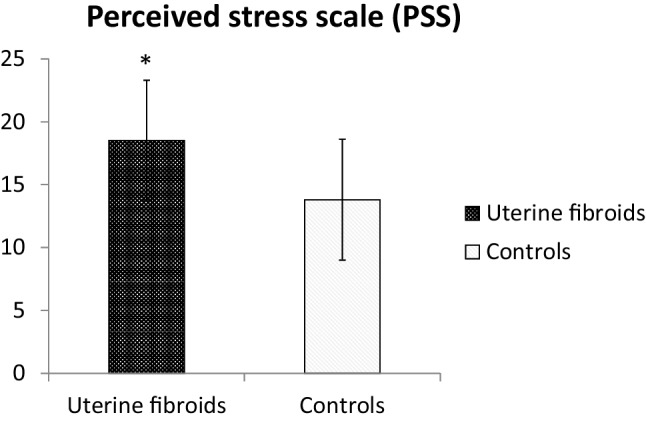
Perceives stress scale among patients with uterine fibroids and controls (18.5 ± 5.0 vs. 13.8 ± 5.0, *p* < 0.001)

Patients with UFs showed significantly higher levels of PSS than controls (18.5 ± 5.0 vs. 13.8 ± 5.0, *p* < 0.001) (Fig. 1). The evaluation of PSS in patients with UFs showed that 59% had medium level of stress, whereas 20% and 21% had high and very high level of stress, respectively. Women with high and very high PSS reported significantly more HMB, severe dysmenorrhea, and impaired social and working life (Table 3).

**Table 3:** Comparison between women with UFs with medium vs high/very high level of stress according to PSS in terms of menstrual cycle characteristics and symptoms

	PSS—medium level of stress (*n* = 38)	PSS—high/very high level of stress (*n* = 31)	*p*
Irregular period	12 (31.6%)	14 (45.1%)	0.220
HMB	18 (47.3%)	26 (83.8%)	0.0024
Pelvic pain	12 (31.5%)	13 (41.9%)	0.453
Pelvic pressure	6 (15.7%)	5 (16.1%)	1
Severe dysmenorrhea	6 (15.7%)	12 (38.7%)	0.05
Severe dyspareunia	3 (7.9%)	3 (9.6%)	1
Impaired working/social life	6 (15.7%)	14 (45.1%)	0.015

**Fig. 2:**
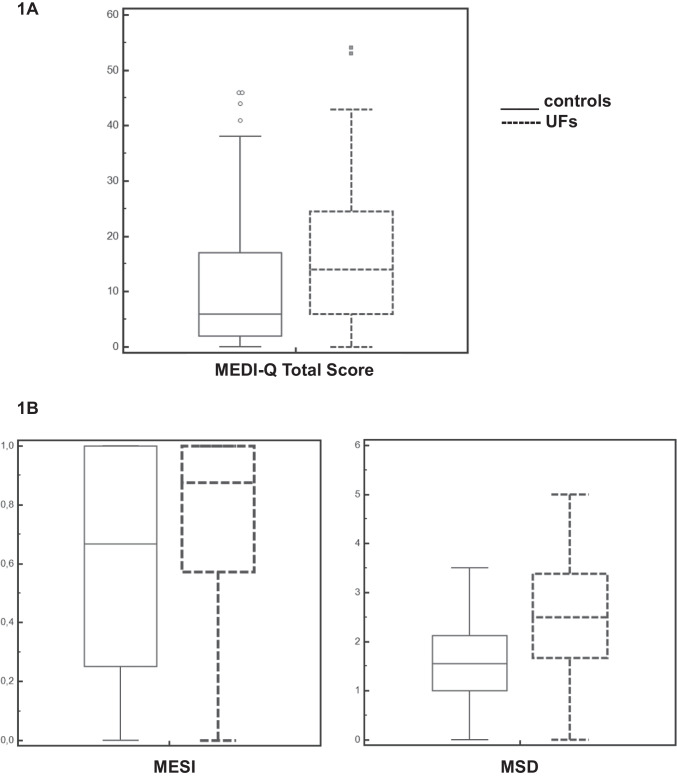
MEDI-Q Total Score (1**A**) and subscales MSD and MESI (1**B**) in patients with UFs and controls

Patients with UFs had significantly higher score for MEDI-Q Total Score (16.51 ± 12.99 vs. 10.86 ± 12.36) (*p* < 0.01) (Fig. 2A) as well as for the subscales MSD (2.54 ± 1.07 vs. 1.57 ± 0.98) (*p* < 0.001) and MESI (0.76 ± 0.30 vs. 0.60 ± 0.39) (*p* < 0.05) (Fig. 2B).

**Fig. 3:**
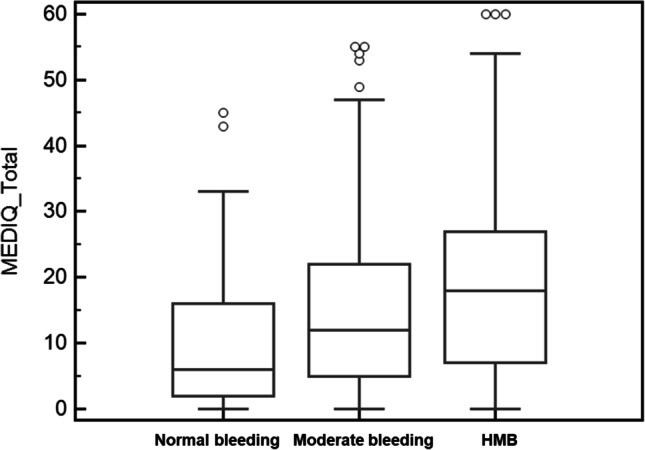
MEDI-Q Total Score according to the amount of menstrual bleeding (normal bleeding 10.36 ± 10.49; moderate bleeding 14.68 ± 12.21; and HMB 19.38 ± 14.39, *p* < 0.0001)

By performing a sub-analysis of each single item determining the global menstrual distress, women with UFs showed a significant distress related to being uncomfortable for menstrual blood loss. Furthermore, lower abdominal pain, nausea, and pain at defecation were found to have a relevant impact in contributing to menstrual distress (Table 4). The relevance of menstrual bleeding as a stressful factor was also supported by the observation in the study population that MEDI-Q Total is significantly higher in patients with heavy bleeding compared to those with moderate and normal bleeding (*p* < 0.0001); similarly, MEDI-Q Total is significantly different in women with moderate bleeding compared to those with normal amount of bleeding (*p* < 0.0001) (Fig. 3). The regression analysis investigating the potential link between the UF characteristics (number, type and size) and the distress scores did not show any significant result.

**Table 4:** Distress score for each item determining the global menstrual distress. ^*^p < 0.05; ^**^p < 0.01; ^***^p < 0.001

Item number	Item description	Controls (*n* = 69)	UFs (*n* = 69)	Statistic
Item 1	Lower abdominal pain	1.07 ± 1.44	1.86 ± 1.90	-2.72^**^
Item 2	Urinary pain	0.04 ± 0.27	0.12 ± 0.56	-0.97
Item 3	Pain at defecation	0.03 ± 0.17	0.19 ± 0.62	-2.05^*^
Item 4	Muscle/osteoarticular pain	0.71 ± 1.32	0.68 ± 1.13	0.14
Item 5	Breast tenderness/widespread swelling sensation	0.46 ± 0.96	0.72 ± 1.21	-1.40
Item 6	Nausea	0.07 ± 0.26	0.58 ± 1.28	-3.23^**^
Item 7	Headache	0.86 ± 1.44	1.20 ± 1.69	-1.30
Item 8	Pain during sexual intercourse	0.03 ± 0.17	0.19 ± 0.90	-1.45
Item 9	Digestive problems	0.19 ± 0.49	0.30 ± 0.83	-1.00
Item 10	Diarrhea	0.36 ± 0.84	0.48 ± 1.09	-0.70
Item 11	Constipation	0.20 ± 0.65	0.23 ± 0.71	-0.25
Item 12	Feeling uncomfortable about vaginal blood loss	0.78 ± 1.37	1.96 ± 1.97	-4.06^***^
Item 13	Feeling of being impure	0.07 ± 0.49	0.22 ± 0.91	-1.17
Item 14	Sadness	0.75 ± 1.35	0.83 ± 1.37	-0.31
Item 15	Emotional lability	0.88 ± 1.29	1.22 ± 1.54	-1.38
Item 16	Irritability/anger	1.17 ± 1.48	1.32 ± 1.59	-0.55
Item 17	Impulsiveness	0.20 ± 0.76	0.49 ± 1.24	-1.65
Item 18	Anxiety	0.33 ± 0.85	0.42 ± 1.01	-0.55
Item 19	Increased appetite	0.58 ± 1.08	0.83 ± 1.48	-1.12
Item 20	Decreased appetite	0.10 ± 0.65	0.10 ± 0.43	0.00
Item 21	Insomnia	0.13 ± 0.77	0.28 ± 0.95	-0.98
Item 22	Hypersomnia	0.25 ± 0.58	0.25 ± 0.81	0.00
Item 23	Fatigue	0.91 ± 1.33	1.10 ± 1.56	-0.76
Item 24	Decreased sexual drive	0.41 ± 0.96	0.49 ± 1.23	-0.46
Item 25	Concentration impairment	0.25 ± 0.88	0.46 ± 1.21	-1.21

## Discussion

The present study showed that patients with UFs have higher level of global perception of stress and menstrual distress compared to healthy controls, as measured by PSS and MEDI-Q, respectively. HMB plays a crucial role in determining such disabling conditions, impairing QoL.

PSS is a measure of the response evocated when an interaction between the environmental factors and the individual is perceived as beyond the resources of the person to manage. The use of this instrument in the UFs patients showed high level of stress, suggesting a perception of insufficient coping resources. Women with the highest levels of perceived stress referred to have HMB and severe dysmenorrhea, confirming the results obtained with the MEDI-Q. The high levels of stress may also explain the high incidence of mental health disorders in patients with UFs, such as anxiety, depression, and panic disorder, which are considered stress-related disorders [25]. Besides, mood disorders and self-directed violence have been shown to be particularly high among women with UFs who experienced pain symptoms or who underwent hysterectomy [29].

The relevant role of menstrual symptoms on stress was supported by the results obtained with the MEDI-Q. In fact, the level of distress resulted to be associated with menstruation-related characteristics, supporting that symptoms interfere with the person’s quality of life, recreational or work activity, and social relationships [26].

Several studies showed a significant impact of UFs on QoL [30, 31], highlighting the burden of UFs symptoms on emotional and psychosocial health [8, 32]. A diagnosis of UFs may be considered per se a relevant psychosocial stressor, with significant patient-reported health disabilities related to bodily pain, mental health, social functioning, and satisfaction with sex life [10]. The instruments normally used for evaluating the QoL are the Short Form-36 (SF-36) or a fibroid-specific symptom and QoL measure, named Uterine Fibroid Symptom and Quality of Life (UFS-QOL) questionnaire [8, 33]. However, these questionnaires are not designed to assess the menstrual distress.

So far, most of the studies have explored the role of stress as a risk factor for UF development [24, 34–37] and not as a consequence. The underlying mechanisms by which stress would be associated with UFs are under discussion [38]. HPA axis hyperactivation may play a role in the genesis of UFs, because it may influence LH and adrenal progesterone secretion, stimulating UFs growth [14]. On primary cultures of human uterine leiomyoma cells [39], it was shown that adrenergic receptor agonists modulate estrogen receptor (ER), progesterone receptor (PR), vascular endothelial growth factor (VEGF), and fibroblast growth factors (FGF), influencing cell proliferation. Moreover, a product of HPA axis, norepinephrine, promotes the synthesis of pro-inflammatory cytokines (e.g., IL (interleukin)-6, IL-1β, IL-10, and tumor necrosis factor-α) involved in the pathogenesis of UFs [38, 40–42].

The present study supports the concept that the presence of UFs contributes to determine distress, as a maladaptive response to the impairment of QoL and of various domains of daily life [23]. The MEDI-Q [26], an instrument designed to assess the menstrual distress, showed significantly higher scores in women with UFs than controls. This measure allows to evaluate the MEDI-Q Total Score, which assesses the global menstruation-related distress, and the MSD and MESI, which measures the distress related to menstrual symptoms and the specificity of menstrual symptoms. Our patients with UFs showed high distress related to the menstruation-related symptoms, as suggested by the indices MSD and MESI, which are specific for the menstruation. HMB is critical in determining this uncomfortable feeling and the amount and duration of menstrual bleeding play the key role in determining menstrual distress. HMB per se is associated with low QoL, because of the resultant anemia and chronic fatigue [43]. Although QoL among women with UFs is significantly impacted by both bleeding and non-bleeding UFs symptoms [6], there is some evidence that menstrual bleeding has the greater effect on QoL than other symptoms [44–46]. Our study showed that the MEDI-Q Total Score raises as the amount of bleeding increases from normal, to moderate, to heavy, further supporting a link between HMB and menstrual distress. Conversely, the UFs number, type and size did not influence the menstrual distress, suggesting the crucial role of symptoms in determining the impact on QoL and stress.

This is the first study to investigate the global perceived stress and the menstruation-related distress in a selected population of women with UFs, according to their symptoms. One of the strengths is the use of a new instrument, the MEDI-Q, which investigates specifically the impact of menstruation on multiple areas, identifying those relevant to determine distress. The study included only women newly diagnosed with UFs, not undergoing any specific medical (hormonal/non-hormonal) or surgical treatment yet, to avoid bias from the therapeutic approach. In addition, cases were matched to controls according to age, in order to get a more homogeneous study population for baseline menstrual pattern related to age itself and parity. However, some limitations should be acknowledged, as the sample size is small and a larger population may be more representative of UF characteristics, in terms of number, size, type, and associated symptoms. In addition, a wider sample would allow to better explore the role of associated gynecological and non-gynecological diseases in contributing to perceived stress. Available evidences show that iron deficiency (ID) has multiple non-erythropoietic effects, including those on mood and neuro-function [47]. Neuro-bioavailability and brain capture of blood iron are necessary for an appropriate synthesis of neurotransmitters (serotonin, dopamine, and noradrenaline) and may explain the effect of ID on mood changes and emotional behavior [48]. Thus, information on health issues related to HMB, such as ID, anemia, and fatigue, may provide further inputs to better understand the link between UF-related symptoms and menstrual distress.

In conclusion, patients with UFs refer significant perceived stress levels and report higher menstrual distress scores compared to controls. On this background, HMB plays a major role in determining the menstrual distress, whereas UF characteristics do not contribute to make this condition as a stressor. Thus, menstruation-related symptoms are key factors to contribute to the global perception of stress among women with UFs.

## Data Availability

The datasets analyzed during the current study are available from the corresponding author on reasonable request.
